# *The Organon* and Materia Medica Club of the Bay Cities of California

**Published:** 1895-11

**Authors:** W. E. Ledyard

**Affiliations:** Secretary


### *THE ORGANON* AND MATERIA MEDICA CLUB OF THE BAY CITIES OF CALIFORNIA.

The regular semi-monthly meeting was held at the office of Dr. W. E. Ledyard, 223 Post Street, San Francisco, July 5th, 1895.

Members present were: Drs. J. M. Selfridge, G. J. Auger, E. W. Bradley, A. McNeil, George H. Martin, M. T. Wilson, and C. M. Selfridge.

The meeting was called to order at 8.15 P. M. by the President, Dr. J. M. Selfridge. The minutes of the previous meeting were read and after a slight correction by Dr. McNeil were approved.

Dr. J. M. Selfridge then said—I would like to relate a case which came to me some time ago from Berkeley, as I think it may be of benefit to any who may hear it. A woman who had dark-red eruption on the face, with pustules here and there, which troubled her a great deal; in fact, more than any other symptom she had. From her history and general condition I deemed it advisable to make a vaginal examination, which I did, and found the womb large and tender, with a protuberance on the left side, which I diagnosed as a fibroid. There were also what I supposed to be adhesions, as I could not move the uterus at all, not even from side to side. The patient had a tubercular tendency, quite a number of her family having died of consumption. I gave her one dose of *Tuberculinum*$^{cm}$, and repeated the dose in the same potency in two weeks. Then in three weeks or a month I gave her Swan's dmm potency. She has taken one or two doses since then. I saw her last Sunday. As she was going away for a few weeks, I said that I would like to make another examination. The first examination had been very painful, and I did not like to examine again, but thought I ought to do it. To my surprise I found that the entire protuberance had disappeared, and also the adhesions, and I could move the uterus easily, though it was about the size of a three-and-a-half months' pregnancy. Hot water douches and the *Tuberculinum* were the only remedies used.

Dr. McNeil—You felt the fibroid clearly?

Dr. J. M. Selfridge—A protuberance was on the side of the uterus, and I thought it a fibroid.

Dr. McNeil—You were sure of the adhesions?

Dr. J. M. Selfridge—There is no doubt about it. I have never supposed that adhesions could be removed by remedies, but now I feel that they can be.

Dr. McNeil then said—I wish to state a point in regard to psora being the cause of post-scarlatinal dropsy, so as not to be misunderstood as to what I said concerning this at a previous meeting. I believe that psora is the *predisposing* cause of post-scarlatinal dropsy, and that cold may be the *exciting* cause.

The President then appointed Dr. McNeil reader of the evening, who read from *The Organon* sections 205 and 206.

DISCUSSIONS.

Dr. McNeil—I wish to give an illustration of this psoric theory. In nearly every case of a boy coming to you with white swelling or hip-joint disease, the parents will tell you that he had a fall some time or other. It is only while the psora is aroused that the injury will cause these conditions. The injury is the *exciting* cause; psora the *predisposing*. In regard to syphilis, there is one sign of suppressed syphilis given by Hahnemann and Zeissl that no other author speaks of, and which is often of great value. A man comes to you and wants to know if he has syphilis; perhaps a little sore appears and heals, but the patient still wonders if he is tainted with syphilis. The point brought out by Hahnemann and Zeissl is this: If the scar of the primary disease is as white as a normal scar would be, there is no syphilis. It will be *blue* if there is syphilis present.

Dr. C. M. Selfridge—Would this include the secondary lesions?

Dr. McNeil—It would also cover a bubo scar. I have several times relieved a man by telling him this, and I think it well to remember.

Dr. Wilson—Is it not possible for a man to have these symptoms without knowing that he has syphilis, having acquired it by heredity?

Dr. McNeil—When you ask this question you speak of the past.

Dr. Wilson—I know that; but I mean, could not all these symptoms be hereditary?

Dr. McNeil—You mean congenital. In such a case you would find the "Hutchinson's teeth." This is the surest guide. By all syphilographers it is given as the main guide.

Dr. C. M. Selfridge—My brother and I had a case of a child with symptoms of inherited syphilis. The notched teeth were present. We knew the grandfather of the child, and knew that he had been wild, and was apt to have syphilis. He was the one who gave it to the child.

Dr. McNeil—Did other symptoms of syphilis develop besides the notched teeth?

Dr. C. M. Selfridge—Yes, several symptoms.

Dr. Wilson—I have frequently found the kind of patients I have just described; patients who were perfectly honest in giving their history, and who had all the symptoms of syphilis, and yet never acquired it through any indiscretion of their own.

Dr. McNeil—Oh! yes; that might be so.

Section 207 was then read.

DISCUSSIONS.

Dr. McNeil—Recently a point has been brought up by Professor Sawyer as regards allopathic drugging. Say Quinine, Mercury, etc., have been given to excess, he gives a high potency as an antidote. Bœnninghausen lays down this fact also, but he was in favor of repeating the dose when desiring to get the antidotal effects. He would ask the patient to succuss it once or twice, he having prepared it in solution, so I would imagine that he gave it once a day. I had a case of lead poisoning in which I found the lead mark on the gums in an old gentleman. *Plumbum*$^{200}$ cured.

Dr. C. M. Selfridge—A case of mine in which an allopathic doctor gave Calomel soon after confinement. The woman's baby was six months old when I saw her. Since the Calomel was taken she had suffered with constipation. I gave her *Mercurius-solubilis*$^{200}$ three hours apart, for two or three weeks, and she was cured. I never gave her anything else.

Dr. McNeil—You traced the constipation to the Calomel?

Dr. C. M. Selfridge—Yes; for she never had been troubled with it before taking the Calomel.

Dr. McNeil—It is rational and in accordance with the principles of Homœopathy to use drugs in this way, and it is well to keep these facts in mind. Hahnemann speaks of the use of Digitalis in heart troubles, and how difficult it is to make a cure when Digitalis has been used. There is usually no hope for such cases. In a recent discussion in Boston Dr. William Wesselhœft stated that he had never seen a case of this kind recover, and that he never takes such a case.

Section 208 was then read.

### Discussions.

Dr. Martin—This section is very important, though it may not appear so in simply reading it. The mental symptoms in any disease are of great importance. If the physician knew the mental symptoms better he could prescribe more accurately. For instance, in constipation, there is a peculiar mental condition present in any person suffering from constipation; they think they must have a movement every day, which is a mental condition which again affects the system, and, in turn, produces more constipation. Allopaths have success because they give remedies to act at once, as a cathartic. Thus the mental symptoms are relieved, and the patient thinks it is wonderful. We sometimes give remedies which relieve constipation in a very short time, and thus the patient's confidence is gained at once, their mental condition is improved, and we effect a cure. If we prescribe simply for the mental symptoms in a case we will be more apt to cure than in any other way.

Dr. Bradley—The question arises with me, is it absolutely necessary to relieve the patient's mind in order to cure? I have always thought that if I gave the right remedy, it would cure in spite of the mental state whether they believe in us or not. When a patient tells me that he hasn't much confidence in homœopathic remedies, I usually tell him that I do not care whether he has or not, that all I want is that he will take my medicines.

Dr. Wilson—Your positive action would bring about the good effect.

Dr. McNeil—I stand by Dr. Bradley. If I can get them to take the medicine, I will take my chances as to cure, and I think they will get better.

Dr. Martin—I think the gentlemen misunderstood me. I

meant if the physician would prescribe for the mental symptoms it would be better. You do not necessarily have to have the faith of your patient in order to perform a cure. I have a case; my own horse suffering from a parasitic skin disease, a sort of mange, for which I prescribed Sulphur. I did not have the faith of my patient in that case, but nevertheless he is getting well. The stable men had been putting on vaseline and other local applications which were rapidly making the case worse. I stopped all these things and depended upon my remedy, with the good result mentioned.

Dr. McNeil—I am happy to agree with Dr. Martin's work. Hahnemann speaks of the mental symptoms being the main ones to prescribe for.

Sections 209–212 were next read.

### Discussions.

Dr. McNeil—A very practical illustration is in our treatment of infants. We are all in the same boat and have felt our weakness in the presence of sick infants. At first sight it seems almost impossible to prescribe for them. Guernsey's *Obstetrics and Diseases of Women and Children* and the last chapters of Hering's *Diseases of the Mind* are two splendid books and they will help us to cure almost every case in infancy. We should learn to study carefully the actions of the babies, as every little sign means something, and there are remedies to meet these various conditions. For instance, the baby wakes cross; raises the very "old Ned" every time he wakes. *Kali-carbonica* and *Lycopodium* are the only remedies for this condition. My own boy put his hands up and says: "Papa is looking at me" or "the girl is looking at me," and there is only one remedy for this and it is *Cina*. Carrying. The child wants to be carried. *Chamomilla* is only one of the drugs for this condition.

Dr. C. M. Selfridge—*Tartar-emetic* is another.

Dr. McNeil—*Pulsatilla* wants to be carried, but must be carried *slowly*. *Tartar-emetic* wants to be carried sitting up. *Cina* wants to be carried, but will scarcely let you touch him; puts his hands up to keep you away. *Arsenic* wants to be carried in a great hurry; "quick, quick." *Veratrum* also wants to be carried in a great hurry. *Borax* will let you carry him, but woe be unto you when you put him down, as he is afraid he will fall. *Cuprum* wants to be carried, but clings to you as he is afraid some one is going to take him. *Bromium* wants to be carried quickly, especially in laryngeal and chest complaints. It is a sort of delirium. *Gelsemium* also clings to you as he is afraid he is going to fall. There are three remedies that don't want to be touched, *Antimonium-crudum, Cina,* and *Tartar-emetic.*

Dr. Martin—*Bryonia* does not want to be touched.

Dr. McNeil—*Bryonia* does not want to be touched because it hurts.

Sections 213–215 were then read.

### Discussions.

Dr. McNeil—I had a case which is a good illustration of the alternation of mental and physical symptoms. Several years ago a lady called upon me, fifty-two years of age, of strong religious faith, very intelligent, and mother of a large family. She prescribed homœopathic remedies herself splendidly, and would have been a credit to the homœopathic profession as a prescriber. Her domestic relations were very happy, and the only cause of her mental condition, a form of melancholia, seemed to be the loss of a grandchild who had died some time previously, after which she had been sick for a long time and lost a great deal of sleep. She made the statement that her menses were coming back, and, as we know, this is always a very suspicious symptom. Dr. Franz Kellar made an examination, and came to the conclusion that incipient cancer was present. An operation upon the cervix, I believe, would have resulted in profound melancholia. She had periodical hemorrhages. The mental symptoms were great suspiciousness, especially of her own family. She thought her children were not treating her right. She wanted to live by herself, and yet thought her daughters were talking about her. She had loose morning stool and was troubled with terrible dreams. I could never get her to tell me what her dreams were about, but she would simply say that they were "disgusting." After a few trials of other remedies, I gave her *Lachesis*, which cured. This was six years ago. She has come twice in two years for more *Lachesis*. She was tottering between cancer and melancholia.

Dr. J. M. Selfridge—Evidently the cancer had not localized itself.

Dr. McNeil—Dr. Kellar said it had. It was a case which would have illustrated the evil effect of the knife. Which is the greater evil, cancer or melancholia?

Dr. J. M. Selfridge—Melancholia is worse than death.

Sections 216–222 were then read.

### Discussions.

Dr. McNeil—Dr. Martin might be able to give us some valuable illustrations upon mental conditions, as he must meet a great deal of it in his special practice.

Dr. Martin—Every thoughtful physician comes to this conclusion: that every abnormal mental condition is preceded by an abnormal physical condition. The majority of neurologists think that the mental state is primary, but I believe there is psora or something of that nature back of it, and that the mental condition is secondary. I had a case of seeming insanity, mania, other physicians having diagnosed it so, and yet I thought it was due to some physical condition and could be cured. Upon a close physical examination, I found the tongue heavily loaded, breath very offensive, many gastric symptoms and considerable sensitiveness in the region of the liver. I concluded that there was a complicating hepatic congestion. *Bryonia* was the remedy, which I gave in the sixth potency, and it was not twenty-four hours before the patient began to improve both in the mental and physical symptoms, and in a week's time the patient was entirely cured.

Dr. J. M. Selfridge—Some years ago I had a case of ulceration of the cervix uteri with suicidal tendencies. There was evidently a psoric basis underlying it. Upon examination there was found an ulceration of the cervix, with endocervicitis. *Aurum* cured.

Dr. Martin—Aurum will always cure where there is a suicidal tendency.

The meeting then adjourned to meet the third Friday in July at the office of Dr. J. M. Selfridge, in Oakland.

Reported by
ELEANOR F. MARTIN, M. D.
(*Stenographer and Typewriter*).

W. E. LEDYARD, *Secretary*.

